# Multiple Health Behavior Changes in a Cancer Prevention Intervention for Construction Workers, 2001-2003

**Published:** 2010-04-15

**Authors:** Amy E. Harley, Carol M. Devine, Binta Beard, Anne M. Stoddard, Mary K. Hunt, Glorian Sorensen

**Affiliations:** Center for Urban Population Health; Cornell University, Ithaca, New York; Harvard School of Public Health, Boston, Massachusetts; New England Research Institutes, Watertown, Massachusetts; Dana-Farber Cancer Institute, Boston, Massachusetts; Harvard School of Public Health, Boston, Massachusetts, and New England Research Institutes, Watertown, Massachusetts

## Abstract

**Introduction:**

Few multiple behavior change interventions have addressed tobacco use in conjunction with fruit and vegetable consumption, particularly among high-risk blue-collar workers. Tools for Health, a cancer prevention intervention for construction laborers, was effective in achieving behavior change for smoking cessation and fruit and vegetable consumption separately. This study examines whether addressing smoking and fruit and vegetable consumption was successful in achieving positive change for both behaviors. We also explored possible explanations for the relationship between behavior changes in these 2 behavioral domains.

**Methods:**

We retrospectively analyzed data from a randomized controlled trial testing a smoking cessation and fruit and vegetable consumption intervention for construction workers. We used survey data from 300 intervention participants to answer our primary research question: Did participants who reported being smokers at baseline successfully quit smoking and increase their fruit and vegetable consumption by the end of the intervention? We used qualitative data from 16 small group discussions to help interpret these results.

**Results:**

Tools for Health participants achieved substantial levels of smoking cessation and increased their fruit and vegetable consumption, concurrently, during the course of the intervention.

**Conclusion:**

This study provides evidence that pairing smoking cessation with increasing fruit and vegetable consumption can be successful in a multiple behavior change intervention designed for high-risk blue-collar workers. Further, our findings provide potential directions for examining why this pairing might be complementary.

## Introduction

Across US occupational classes there are growing disparities in disease risk. Illnesses and deaths associated with chronic diseases are partially attributable to multiple health behaviors, including diet and smoking (1-3). A recent study reported that the prevalence of smoking among blue-collar workers was 35%, which is double that of white-collar workers (4). This finding corroborates previous studies regarding the disparity in smoking rates by occupational class (5). Although blue-collar workers attempt to quit smoking at similar or higher rates than other occupational classes, evidence indicates that declines in smoking prevalence are slower and rates of successfully quitting are lower than those for white-collar workers (4-6). Dietary patterns illustrate another health disparity: blue-collar, low-wage workers are less likely to eat healthfully or to consume the recommended daily number of fruits and vegetables than are white-collar workers (7).

Worksite programs play a pivotal role in addressing disparities in health behaviors by occupational class. Most research-based worksite interventions, however, adopt a single-behavior approach (2,3). Research indicates that higher risks for cardiovascular disease and cancer are associated with a combination of behaviors that tend to cluster in people and populations (8-10). Moreover, research demonstrates that smokers eat fewer servings of fruits and vegetables than do nonsmokers (11,12). High rates of smoking and low rates of fruit and vegetable consumption among blue-collar workers, paired with the evidence of behavior clusters, suggest that targeting these behaviors in 1 intervention could offer participants the opportunity to quit smoking and improve their diet concurrently.

Few multiple health behavior change intervention studies have addressed tobacco use in conjunction with fruit and vegetable consumption (13-17). The available studies have addressed behaviors in addition to tobacco use and fruit and vegetable consumption. To our knowledge, only 1 study examined relationships between tobacco use and fruit and vegetable consumption, reporting that stages of change for fruit and vegetable consumption and smoking cessation were separate constructs (14).

Tools for Health (TFH) was a randomized, controlled trial that tested a cancer prevention intervention for construction workers. The intervention targeted smoking cessation and fruit and vegetable consumption using tailored telephone counseling. Primary findings from TFH, reported in 2007, were that the intervention was effective in achieving statistically significant increases in smoking cessation and fruit and vegetable consumption separately among blue-collar workers (18).

TFH was conducted in collaboration with the Laborers' International Union of North America (LIUNA). LIUNA represents approximately 400,000 construction workers in the US and Canada (18). According to the Bureau of Labor Statistics, about 15% of workers in the construction trades were union members (19). This unionization rate is somewhat higher than for other industries. TFH participants most often reported working in the following job categories: general laborer (29%), concrete worker (19%), heavy construction worker (19%), demolition worker (12%), and jackhammer (12%) (18).

The primary objective of this investigation was to examine change across the 2 behaviors. Specifically, we sought to determine whether smokers in TFH changed both their smoking and dietary behaviors. The secondary objective was to explore possible explanations for the relationship between changes in 2 behavioral domains: smoking and fruit and vegetable consumption. Our hypothesis was that participants who quit smoking during the intervention would not report an increase in fruit and vegetable consumption.

## Methods

For this article we examined quantitative data collected from telephone and mail surveys, administered as a background survey and baseline and final efficacy surveys. Participants represented a national sample of US LIUNA workers. We also examined discussion  group data that were collected by the TFH research team for the formative phase of the study. We used the survey data to evaluate the primary question: Did smokers at baseline successfully quit smoking and increase their fruit and vegetable consumption? We used the qualitative data to help interpret these results. The institutional review board of the Dana-Farber Cancer Institute approved the study and participants signed informed consent forms.

### Background survey

The background survey, conducted from May 2001 through November 2002, provided population-based data, including social contextual factors and sociodemographic data. LIUNA provided contact information for 3,239 members randomly selected from the union roster. Of those, 1,005 could not be contacted. Members were eligible to participate if their membership was current, they were not retired or on disability, were working in construction, and could complete the survey in English or Spanish. Researchers reached 2,234 workers, of whom 477 (21%) were ineligible to participate because they did not meet the inclusion criteria. Assuming 21% (215) of those not contacted were ineligible, we estimate the total number of ineligibles to be 692. Thus, we attempted to contact 2,547 eligible workers.

A total of 1,108 of the 2,547 eligible respondents completed the survey (44% response rate). To calculate the response rate to the background survey, we assumed that 21% (215) of those we were unable to contact were also ineligible (477/2,234 = 21%). Thus, we estimate the total number of ineligibles to be 692 (477 + 215) and the total eligible to contact to be 2,547 (3,239 − 692). The response rate among eligible members was 44% (1,108/2,547). We collected 1,109 surveys; 1 survey was excluded because of incomplete data.

Sociodemographic variables including sex, race/ethnicity, educational level, and native language were measured. We also assessed work/family spillover, a term used to describe work roles affecting participation in family roles. We assessed this construct by using a 6-item scale (Cronbach's α = 0.74) (21). In this scale, respondents were asked to rate their agreement with statements about their work negatively affecting their family (eg, missing meals or feeling distracted at home because of work).

### Baseline and final efficacy surveys

We invited the 1,108 background survey respondents to participate in the randomized trial from November 2002 through December 2003. Of the 915 that remained eligible (194 retired or had lapsed memberships), 673 (74%) consented to participate, completed the baseline survey, and were randomized into the study. Six months later, 582 participants (86%) completed the final survey. Data collection was completed in July 2004.

Cigarette use was determined by self-report on the basis of recommendations by the Society for Research on Nicotine and Tobacco (21). Smoking cessation was measured by the prevalence of smokers and recent quitters (in the last 6 months) at baseline who reported not smoking on the final survey. Fruit and vegetable consumption was measured with a screening tool used in the National Cancer Institute's 5-A-Day for Better Health projects (22). Correlations between intake measured by 24-hour diet recall and the all-day screener have been estimated at 0.66 for men and 0.51 for women (23). The following food groups were included: 100% fruit juice, lettuce salads, fried potatoes, other white potatoes, cooked dried beans, fruits, and other vegetables. Total servings of fruits and vegetables per day were computed by summing the servings per day of each, excluding fried potatoes. Additional variables measured on the baseline survey included marital status, number of minor children in the household, intention to change fruit and vegetable consumption, and whether eating at work helps cope with stress.

We used data from the 300 participants who were randomized to the intervention group and completed the final survey. Analysis began with the creation of a new smoking status variable with 3 categories: nonsmoker (nonsmoker at baseline and final), smoker (smoker at final irrespective of baseline smoking status), and quitter (smoker at baseline, not final). We then examined the relationship between this smoking status variable and change in fruit and vegetable consumption by using analysis of covariance. To explore potential confounders to this relationship, we examined relationships between change in fruit and vegetable consumption and variables including sex, race/ethnicity, education, native language, living with a partner, children in household, work/family spillover, work hours, intention to increase fruit and vegetable consumption, and eating at work to cope with stress, controlling for baseline consumption.

We then constructed a multivariable model examining the relationship between smoking status and change in fruit and vegetable consumption, controlling for all variables individually associated with change in consumption. Significance was set at *P* < .05. We added all variables into the model as a group. The final model assessed the relationship between smoking status and change in fruit and vegetable consumption, controlling for baseline consumption and all potential confounding variables individually associated with change in consumption. Analyses were conducted by using SAS statistical software version 9.1 (SAS Institute, Inc, Cary, North Carolina).

### Small group discussions

Discussion groups were originally conducted during the formative phase of TFH (November 2001-April 2002) (24). Because each discussion group included discussion about smoking and fruit and vegetable consumption, we examined these data to help interpret the survey findings by gaining insight into the links between these 2 behaviors from the workers' perspective.

We recruited a nationally representative sample of LIUNA members, through LIUNA's nationwide training centers, using purposive stratified sampling (25). The sampling strategy was constructed to ensure variation in the overall sample on the following variables: smoking status, sex, race/ethnicity, and geographic region. The final sample included 88 participants in 16 discussion groups (median number of participants per group, 5; range, 3-9).

We designed 2 semistructured topic guides to facilitate the groups. The first was used to explore members' perceptions of being a laborer, the union, work relationships, and health-related concerns. The second, used after prototype intervention materials were developed, was used to elicit participants' reactions to these materials. All sessions were recorded and transcribed. Although the primary purpose of the discussion groups was formative, both topic guides elicited discussion around experiences with smoking and healthy eating, yielding data relevant to this investigation.

The purpose of the group analysis was to explore relationships between smoking cessation and fruit and vegetable intake. Data analysis began by reading each transcript. Codes were applied to transcripts in QSR NVIVO version 1.2 (QSR International, Inc, Cambridge, Massachusetts) by using a 2-stage coding process: deductive coding followed by thematic (inductive) coding. For deductive coding, a codebook was developed to classify data into 3 topics: diet, smoking, and experiences/beliefs about behavior change. Data in each segment were further analyzed by using thematic coding, which explored themes that linked dietary habits with smoking habits or behavior change experiences or beliefs that linked the 2 behaviors. No a priori codebook was used during this stage. The first author (AH) assigned codes to emergent themes, queried codes, and reviewed and summarized the data.

## Results

### Survey data

Participants were mostly male, non-Hispanic whites, and had completed a high school diploma or equivalent (Table 1). Participants varied by the smoking status groups on 2 factors: native language and intention for changing fruit and vegetable consumption. A comparison in language groups suggests that more participants whose first language was not English were nonsmokers (86%) than were their peers whose first language was English (63%). Nonsmokers also reported higher levels of intention to increase fruit and vegetable consumption than did smokers at baseline.

The relationship between smoking status and change in fruit and vegetable consumption controlling for baseline intake was significant (*P* = .02) (data not shown). Nonsmokers and quitters showed an average increase in fruit and vegetable consumption of 1.92 servings (95% confidence interval [CI], 1.37-2.45) and 2.47 (95% CI, 0.75-4.19) compared with smokers, who increased their mean fruit and vegetable consumption by 0.58 servings (95% CI, −0.22 to 1.38).

We examined the association of each potential confounding variable with mean change in fruit and vegetable intake, controlling for baseline intake (Table 2). Sex, education, native language, eating at work to cope with stress, and intention to change fruit and vegetable consumption exhibited relationships with change in fruit and vegetable consumption. We compared the multivariable model containing smoking status with the significant variables (Table 2), controlling for baseline intake (Table 3). When other variables were controlled, fruit and vegetable intention and smoking status remained significant. The *R* ^2^ for this model was 0.15.

In the multivariable model, we suspected that fruit and vegetable intention might be over-controlling, so we removed it. As in the full model, smoking status remained a significant predictor of change in fruit and vegetable consumption. Overall, the relationships with the remaining variables changed little and the *R* ^2^ decreased to 0.12.

### Discussion group data

Like the intervention participants, the 88 discussion group participants were primarily white non-Hispanic (64%), men (84%), and reported completing high school or equivalent (54%). Seventy-eight percent of discussion group participants identified as current smokers. They ranged in age from 20 to 63 years (24). Four themes emerged that potentially linked smoking cessation with increased fruit and vegetable consumption: 1) concern for overall health, 2) concern about weight gain, 3) taste preferences, and 4) behavioral compensation, specifically, the need for quitters to keep their hands busy.

Group participants presented improving overall health as a reason for wanting to quit smoking and improve eating habits. In response to questions about why participants might want to eat more fruits and vegetables, they said, "Eating better will make you healthier," and "It's healthier food than anything, even the meats."  Responses to parallel questions about why participants wanted to or had quit smoking included, "I quit because I know it's bad for my health," and "That's why I decided I needed to let [smoking] go because it wasn't making my health no better." One explanation for why quitters changed both smoking behaviors and fruit and vegetable consumption during the same intervention is that changing both behaviors targets overall health improvement.

The second theme linking smoking cessation with fruit and vegetable consumption was concern with weight gain during smoking cessation. In discussions about desire to quit smoking, workers mentioned being worried about gaining weight if they quit. Participants' statements included, "Everybody that quits smoking gains weight," and

My doctor told me you gain an average of 20 pounds when you quit smoking. So I went on a diet and lost 20 pounds. Eight weeks later, I not only gained the 20 pounds back when I quit smoking, but also another 16. You betcha I went back to smoking.

Another theme that linked smoking with fruit and vegetable consumption among those with previous quit attempts was that food tasted better during the quit attempt. Participants said, "Food tastes better," "You can't even taste food when you smoke. When you quit smoking for 2 or 3 weeks, you can taste the food better already. Cigarettes take away taste," and "Food even tastes completely different when you quit." This theme may indicate that when someone is not smoking, improvement in fruit and vegetable taste may promote increased consumption.

The fourth theme that linked smoking with fruit and vegetable consumption was behavioral compensation. Discussion group participants discussed needing something to do with their hands during quit attempts. Sample comments include, "That's part of the whole pattern, is the handling," "You quit smoking. What do you do with your hands?" and "I don't smoke for the nicotine. I smoke to have something to do with my hands." Because fruit and vegetable consumption requires handling, especially preparing and eating raw fruits and vegetables, increasing consumption might have offered a substitute for keeping a smoker's hands busy during smoking cessation.

## Discussion

The TFH intervention was associated with significant change in smoking cessation and fruit and vegetable consumption among construction workers. Although the workers who quit smoking substantially increased their fruit and vegetable consumption, it remains unclear why these 2 behaviors were related. Our discussion group data highlight 4 possible mechanisms linking these 2 behaviors: overall health, concern about weight gain, taste, and behavioral compensation.

Our key finding is that TFH effectively increased smoking cessation and fruit and vegetable consumption concurrently. Other studies intervening on smoking and fruit and vegetable consumption through a single intervention did not show significant change in both behaviors (13,15,16). Only 1 of these studies assessed change across behaviors (16). It reported significant change in a multiple risk-factor index. However, this study did not discern whether changes in smoking status and fruit and vegetable intake were related to each other or to changes in any of the other health behaviors assessed.

The findings from our study help to fill gaps in knowledge about the efficacy of addressing smoking and fruit and vegetable consumption in a multiple health behavior change intervention. These findings provide support for designing and implementing interventions that address 2 key health behaviors among high-risk blue-collar workers. Blue-collar workers' high rates of smoking and low rates of fruit and vegetable consumption illustrate the urgent need for interventions that can reduce socioeconomic and occupational disparities in chronic disease risk.

Several studies support the discussion group themes on pairing smoking cessation with fruit and vegetable consumption. One study showed that 26% of male smokers were concerned about weight (26). Men interested in controlling their weight during quit attempts may be predisposed to attend to messages regarding low-calorie food alternatives. At least 1 study demonstrated that cigarettes worsen the taste of fruits and vegetables (27). If the smoking cessation portion of the intervention precedes dietary changes, the poor taste of fruits and vegetables due to cigarette smoking would no longer serve as a disincentive to consumption. Finally, we found no studies that explicitly explored fruit and vegetable consumption as a substitute for handling cigarettes. However, this strategy is evident in published tips for quitting smoking (28). Further examination of the mechanisms by which these behaviors may complement each other is necessary to assess the usefulness of their pairing in multiple health behavior change interventions.

Although the findings from this study contribute new insights on multiple health behavior change, limitations should be noted. The response rate to the background survey was low. These results should be generalized only to those who meet the eligibility criteria of the study. However, because of randomization at enrollment, internal validity is assured. The size of the quitter group was small. In addition, survey data were limited on the process of behavior change. It was not possible to thoroughly examine quantitatively why this pairing was efficacious.

This study provides evidence that pairing smoking cessation with increasing fruit and vegetable consumption can be successful in a multiple health behavior change intervention designed to address high-risk blue-collar workers. Furthermore, our findings provide some potential directions for examining why this pairing might be complementary. As the process and measurement of multiple health behavior change interventions gains further attention (3), empirical evidence is necessary to guide the selection of behaviors for inclusion in these interventions.

## Figures and Tables

**Table 1:** Baseline Characteristics of Intervention Participants by Smoking Status, LIUNA, 2001-2003 (N = 300)

Characteristic	Nonsmoker^a^,^b^, n (%)	Smoker^c^, n (%)	Quitter^d^, n (%)	***P* Value^e^**
**Sex **				
Men	180 (95)	81 (91)	18 (100)	.19
Women	9 (5)	8 (9)	0	
**Race/ethnicity**				
Hispanic	32 (17)	6 (7)	4 (21)	.20
Non-Hispanic white	127 (66)	71 (79)	11 (58)	
Non-Hispanic black	22 (12)	7 (8)	3 (16)	
Other	10 (5)	6 (7)	1 (5)	
**Education**				
Less than high school diploma	31 (16)	19 (21)	6 (32)	.60
High school diploma or GED	86 (45)	42 (47)	7 (37)	
Post-high school training	67 (35)	25 (28)	5 (26)	
Bachelor's degree or higher	6 (3)	4 (4)	1 (5)	
**Native language**				
English	132 (87)	65 (98)	12 (86)	.03
Other	19 (13)	1 (2)	2 (14)	
**Married or living with partner **				
Yes	147 (77)	72 (80)	16 (84)	.68
No	44 (23)	18 (20)	3 (16)	
**Children in household **				
Yes	107 (56)	50 (56)	7 (37)	.29
No	84 (44)	40 (44)	12 (63)	
**Work/family spillover^f^ **				
High	21 (12)	14 (16)	2 (11)	.62
Medium	70 (40)	35 (41)	5 (28)	
Low	85 (48)	37 (43)	11 (61)	
**Hours worked weekly**				
Part-time (<38)	9 (5)	6 (7)	0	.29
Full-time (38-42)	103 (54)	43 (48)	14 (74)	
Overtime (>42)	79 (41)	41 (46)	5 (26)	
**Intention to change fruit and vegetable intake^g^**				
Precontemplation	72 (38)	51 (57)	10 (53)	.05
Contemplation	12 (6)	5 (6)	1 (5)	
Preparation	106 (56)	34 (38)	8 (42)	
**Eat at work to cope with stress**				
Agree	111 (58)	46 (51)	15 (79)	.15
Disagree	79 (41)	42 (47)	4 (21)	
No opinion/missing	1 (1)	2 (2)	0	

Abbreviations: LIUNA, Laborers' International Union of North America; HS, high school; GED, General Educational Development certificate.

a Nonsmokers reported not smoking in the previous 7 days at both the baseline and final surveys.

b Column totals do not always sum to 300 because of missing values for individual items.

c Smokers reported smoking in the previous 7 days at the final survey irrespective of baseline smoking.

d Quitters reported smoking in the previous 7 days at baseline, but not smoking at final survey.

e *P* value for exact test of hypothesis of homogeneity of distributions.

f Extent to which work role interferes with family role.

g Intention to change is measured by the stages of change model. People in the precontemplation stage have the lowest intention to change fruit and vegetable intake.

**Table 2:** Associations Between Individual Explanatory Variables and Change in Fruit and Vegetable Consumption, Controlling for Baseline Consumption, LIUNA, 2001-2003

**Variable**	Mean Change in Consumption (95% CI)	*P* Value^a^
**Sex**		
Men	1.61 (1.16 to 2.06)	.04
Women	−0.36 (−2.18 to 1.46)	
**Education**		
Less than high school diploma	2.86 (1.86 to 3.89)	.005
High school diploma or GED	1.34 (0.69 to 1.99)	
Post-high school training	0.83 (0.07 to 1.59)	
Bachelor's degree or higher	3.31 (1.06 to 5.56)	
**Native language **		
English	1.19 (0.72 to 1.66)	.001
Other	3.79 (2.63 to 4.95)	
**Eat at work to cope with stress **		
Agree	1.94 (1.37 to 2.51)	.03
Disagree	0.94 (0.27 to 1.61)	
**Intention to change fruit and vegetable intake**		
Precontemplation	0.64 (−0.01 to 1.29)	.001
Contemplation	3.38 (1.64 to 5.07)	
Preparation	2.09 (1.48 to 2.70)	
**Smoking status**		
Nonsmoker^b^	1.92 (1.37 to 2.47)	.007
Smoker^c^	0.45 [(−0.35 to 1.25)	
Quitter^d^	2.47 (0.75 to 4.19)	
**Race/ethnicity**		
Hispanic	2.65 (1.47 to 3.83)	.12
White only	1.24 (0.71 to 1.77)	
Black only	2.10 (0.77 to 3.43)	
Other	0.93 (−0.89 to 2.75)	
**Weekly hours worked**		
Part-time (≤37)	1.19 (−0.75 to 3.13)	.18
Full-time (38-42)	1.90 (1.29 to 2.51)	
Overtime (>42)	1.06 (0.39 to 1.73)	
**Married or living with partner**		
No	0.97 (0.03 to 1.91)	.21
Yes	1.66 (1.17 to 2.15)	
**Work/family spillover^f^**		
Low	1.48 (0.83 to 2.13)	.68
Medium	1.71 (0.98 to 2.44)	
High	1.09 (−0.14 to 2.32)	
**Children in household**		
No	1.45 (0.81 to 2.10)	.79
Yes	1.56 (0.97 to 2.15)	

Abbreviations: LIUNA, Laborers' International Union of North America; CI, confidence interval; General Educational Development certificate.

a *P* value for test of equality of mean change, controlling for baseline consumption.

b Nonsmokers reported not smoking in the previous 7 days at both the baseline and final surveys.

c Column totals do not always sum to 300 because of missing values for individual items.

d Smokers reported smoking in the previous 7 days at the final survey irrespective of baseline smoking.

e Quitters reported smoking in the previous 7 days at baseline, but not smoking at final survey.

f Extent to which work role interferes with family role.

**Table 3:** Multivariable Association of Smoking Status With Change in Fruit and Vegetable Consumption, Controlling for Potential Covariates, LIUNA, 2001-2003

** Variable**	Adjusted Mean Change in Consumption** (95% CI)**	*P* Value^a^
**Sex **		
Men	2.93 (1.91 to 3.95)	.30
Women	1.94 (−0.14 to 4.02)	
**Education **		
Less than high school diploma	2.71 (1.18 to 4.24)	.11
High school diploma or GED	1.72 (0.31 to 3.03)	
Post-high school training	1.48 (−0.03 to 2.99)	
Bachelor's degree or higher	3.83 (1.30 to 6.36)	
**Native language **		
English	0.89 (−0.42 to 2.20)	.17
Other	2.97 (1.21 to 4.73)	
**Eat at work to cope with stress **		
Agree	2.70 (1.25 to 4.42)	.25
Disagree	2.17 (0.76 to 3.58)	
**Intention to change fruit and vegetable intake **		
Precontemplation	1.31 (−0.04 to 2.66)	.02
Contemplation	3.77 (1.63 to 5.91)	
Preparation	2.2 (0.85 to 3.59)	
**Smoking status **		
Nonsmoker^b^	2.93 (.60 to 4.26)	.04
Smoker^c^	1.69 (0.24 to 3.14)	
Quitter^d^	2.69 (0.61 to 4.77)	

Abbreviations: LIUNA, Laborers' International Union of North America; CI, confidence interval; General Educational Development certificate.

a *P* value for test of equality of mean change controlling for all other covariates and baseline consumption.

b Nonsmokers reported not smoking in the previous 7 days at both the baseline and final surveys.

c Smokers reported smoking in the previous 7 days at the final survey irrespective of baseline smoking.

d Quitters reported smoking in the previous 7 days at baseline, but not smoking at final survey.
